# Prognostic significance of macrophage invasion in hilar cholangiocarcinoma

**DOI:** 10.1186/s12885-015-1795-7

**Published:** 2015-10-24

**Authors:** Georgi Atanasov, Hans-Michael Hau, Corinna Dietel, Christian Benzing, Felix Krenzien, Andreas Brandl, Georg Wiltberger, Ivan Matia, Isabel Prager, Katrin Schierle, Simon C. Robson, Anja Reutzel-Selke, Johann Pratschke, Moritz Schmelzle, Sven Jonas

**Affiliations:** 1 Department of General, Visceral and Transplantation Surgery and Department of General, Visceral, Vascular and Thoracic Surgery, Charité – Universitätsmedizin Berlin, Charitéplatz 1, 10117 Berlin, Germany; 2 Department of Visceral-, Transplantation-, Thoracic- and Vascular Surgery, University Hospital Leipzig, Leipzig, Germany; 3 Institute of Pathology, University Hospital Leipzig, Leipzig, Germany; 4 The Transplant Institute and Division of Gastroenterology, Beth Israel Deaconess Medical Center, Harvard University, Boston, MA USA; 5 Translational Centre for Regenerative Medicine, Leipzig University, Leipzig, Germany; 6 Centre Hépato-Biliaire, Université Paris Sud, Hôpital Paul Brousse, Paris, France

**Keywords:** Hilar cholangiocarcinoma, Tumor associated macrophages, TAMs, CD68, Liver resection

## Abstract

**Background:**

Tumor-associated macrophages (TAMs) promote tumor progression and have an effect on survival in human cancer. However, little is known regarding their influence on tumor progression and prognosis in human hilar cholangiocarcinoma.

**Methods:**

We analyzed surgically resected tumor specimens of hilar cholangiocarcinoma (*n* = 47) for distribution and localization of TAMs, as defined by expression of CD68. Abundance of TAMs was correlated with clinicopathologic characteristics, tumor recurrence and patients’ survival. Statistical analysis was performed using SPSS software.

**Results:**

Patients with high density of TAMs in tumor invasive front (TIF) showed significantly higher local and overall tumor recurrence (both *ρ* < 0.05). Furthermore, high density of TAMs was associated with decreased overall (one-year 83.6 % vs. 75.1 %; three-year 61.3 % vs. 42.4 %; both *ρ* < 0.05) and recurrence-free survival (one-year 93.9 % vs. 57.4 %; three-year 59.8 % vs. 26.2 %; both *ρ* < 0.05). TAMs in TIF and tumor recurrence, were confirmed as the only independent prognostic variables in the multivariate survival analysis (all *ρ* < 0.05).

**Conclusions:**

Overall survival and recurrence free survival of patients with hilar cholangiocarcinoma significantly improved in patients with low levels of TAMs in the area of TIF, when compared to those with a high density of TAMs. These observations suggest their utilization as valuable prognostic markers in routine histopathologic evaluation, and might indicate future therapeutic approaches by targeting TAMs.

## Background

Hilar cholangiocarcinoma represents the most common cancer arising within the extrahepatic bilary tree and extended liver resection or liver transplantation following a highly selective protocol with combined neoadjuvant radiochemotherapy represent the only curative treatment [1]. High risk of tumor recurrence remains a serious problem, even if liver resection is combined with extrahepatic hilar en bloc resection [2, 3]. The seventh edition of the TNM classification separates extrahepatic bile duct tumors into perihilar and distal tumors [4]. Modifications of staging systems for hilar cholangiocarcinoma in order to enhance prognostic accuracy have recently been proposed [5, 6]. There is an urgent need to identify prognostic markers associated with recurrence and survival. A better understanding of underlying biological mechanisms might further help to improve treatment options in this tumor entity.

All classes of leukocytes are found within malignant tumors. Tumor-associated macrophages (TAMs) constitute up to 50 % of this leukocyte cell population. Monocytes are recruited from the circulation at sites of injury, inflammation, infection, and malignancy, where they differentiate into tissue macrophages [7–10]. TAMs are diffusely found throughout tumorous tissue in localized zones, e.g. tumor invasive fronts (TIF), around ductal and in tumor stromal areas [11, 12]. Experimental data have highlighted a fundamental role of TAMs in tumor progression [13]. High abundance of TAMs is associated with an unfavorable prognosis in hepatocellular carcinoma (HCC), esophageal, ovarian and breast cancer and recent studies have emphasized a link between their abundance in tumor tissues and the process of tumor spread [14–19].

The clinical significance of infiltrating TAMs remains uncertain in hilar cholangiocarcinoma. The aim of this study was therefore to evaluate the relationship between abundance of TAMs and a presumed association with tumor growth, metastasis, recurrence and clinical prognosis in hilar cholangiocarcinoma.

## Methods

### Patients and tumor samples

A total of 47 patients who underwent major hepatectomy between January 1996 and December 2002 for hilar cholangiocarcinoma were included in the study. Hilar cholangiocarcinoma was confirmed histopathologically and classified according to the American Joint Committee on Cancer/Union Internationale Contra Cancrum tumor-node-metastasis classification (UICC) classification. Written informed consent was obtained from all patients. This study was approved by the ethics committee of Charité – Universitätsmedizin Berlin.

In all patients liver resection was in curative intent. None of the patients received neoadjuvant radio- and/or chemotherapy prior to surgery. None of the patients died in the postoperative course. In 37 of 47 (78.7 %) patients a curative resection was accomplished (histopathologically confirmed negative resection margin; R0 status), in 5 patients (10.6 %) R1 status was diagnosed and in another 5 (10.6 %) patients R2 situation was pathologically confirmed.

Formalin-fixed, paraffin-embedded tumor samples were retrieved from the files of the Institute of Pathology. Tissue blocks embedding a representative sample of the tumor were used. Histological diagnosis of the primary tumor stage and nodal status were determined by hematoxylin and eosin (H&E) stained sections. The clinicopathological characteristics of the study population are depicted in Table 1.

**Table 1:** Clinicopathological characteristics of the patients included in the study

Clinicopathological characteristics	ᅟ
Variable	Value (%)
No. of patients	47
Gender	
Male	23 (48.9 %)
Female	24 (51.1 %)
Patient age	
≤60	23 (48.9 %)
>60	24 (51.1 %)
Histologic differentiation	
Well/Moderate	40 (85.1 %)
Poor	7 (14.9 %)
Pathologic T stage	
T2	16 (34.0 %)
T3	31 (66.0 %)
Pathologic N stage	
Negative	32 (69.1 %)
Positive	15 (31.9 %)
Perineural sheath infiltration	
Negative	4 (8.5 %)
Positive	43 (91.5 %)
Lymphangiosis carcinomatosa	
Negative	25 (53.2 %)
Positive	22 (46.8 %)
Perivascular lymphangiosis	
Negative	24 (51.1 %)
Positive	23 (48.9 %)
Vascular invasion	
Negative	32 (68.1 %)
Positive	15 (31.9 %)
Tumor recurrence	
Without	22 (46.8 %)
With	25 (53.2 %)
Local recurrence	
Without	27 (57.4 %)
With	20 (42.6 %)
Distant metastases	
Without	36 (76.6 %)
With	11 (23.4 %)

### Immunohistochemistry

Formalin-fixed and paraffin-embedded tumor sections (5 μm thick) were dewaxed and rehydrated. Antigen retrieval was performed by heating the slides in 10 mM Tris buffer with 1 mM EDTA (pH 9) in a streamer for 20 min. Endogenous peroxidase activity was inhibited with 3 % H_2_O_2_ for 5 min. After washing with Tris buffered saline (TBS) with tween, the endogenous biotin was suppressed by sequential incubations with 0,1 % avidin and 0,01 % biotin (Dako, Glostrup, Denmark) for 10 min each at room temperature. Additional nonspecific binding sites were blocked with 3 % skimmed milk powder for 30 min at room temperature. Tissue sections were incubated with the monoclonal mouse antibody anti-human CD68 Clone PG-M1 (1:50, Dako, Glostrup, Denmark) for 30 min at room temperature. The universal LSAB+ system-HRP (Dako, Glostrup, Denmark) and the DAB+ liquid substrate chromogen system (Dako, Glostrup, Denmark) was applied for the visualization of the antibody reaction. The biotinylated anti-rabbit, anti-mouse and anti-goat immunoglobulin and the streptavidin conjugated horseradish peroxidase of the universal LSAB+ system was incubated consecutively for 20 min each. Sections were counterstained with hematoxylin. Specificity controls were performed without primary antibody.

### Quantification of CD68 density

All specimens were evaluated by three independent researchers (GA, CD and IP), and an independent pathologists (KS), without any knowledge of prognosis or clinicopathological variables. TAMs were defined by their expression of CD68. For quantification of infiltrating TAMs the whole tumor area was thoroughly investigated for presence of CD68-positive cells. The evaluation of TAMs density was performed as percent immunohistochemical CD68 staining related to tumor cell amount.

The density of TAMs in the whole tumor area (tumorous tissue, TT) was semi-quantitatively classified into the following categories: 0, negative; 1, 1–25 %; 2, 26–75 %; and 3, >75 %. For statistical analysis, TT scores 0 and 1 were categorized as low density, and TT scores 2 and 3 as high density of TAMs. The density of TAMs in the tumor infiltrating front (TIF) were semi-quantitatively classified into the following major categories: 0, negative; 1, 1–25 % and 2, >25 %. For statistical analysis, TIF scores 0 and 1 were categorized as low density or negative, TIF score 2 as high density (or positive).

Patients with hilar cholangiocarcinoma were divided into several groups either by ‘low’ or ‘high’ density of macrophages in tumorous tissue (‘low TT CD68 group’; *n* = 24) and ‘high TT CD68 group’; *n* = 23, respectively), or ‘low’ or ‘high’ density of TAMs in TIF (‘low TIF CD68 group’ *n* = 21; and ‘high TIF CD68 group’, *n* = 26, respectively).

### Statistical analysis

Survival analysis, univariate analysis and Kaplan-Meier curves were generated with assistance of the SPSS software program (Version 19.0.0 / Year 2010). Comparison of categorical and continuous variables was performed using the Chi^2^-test and the Wilcoxon-test, respectively. Survival data were compared with the log rank-test. Variables with a significant influence on survival in the univariate analysis were entered into a cox regression analysis. A difference was considered significant for *p* < 0.05.

## Results

**Fig. 1:**
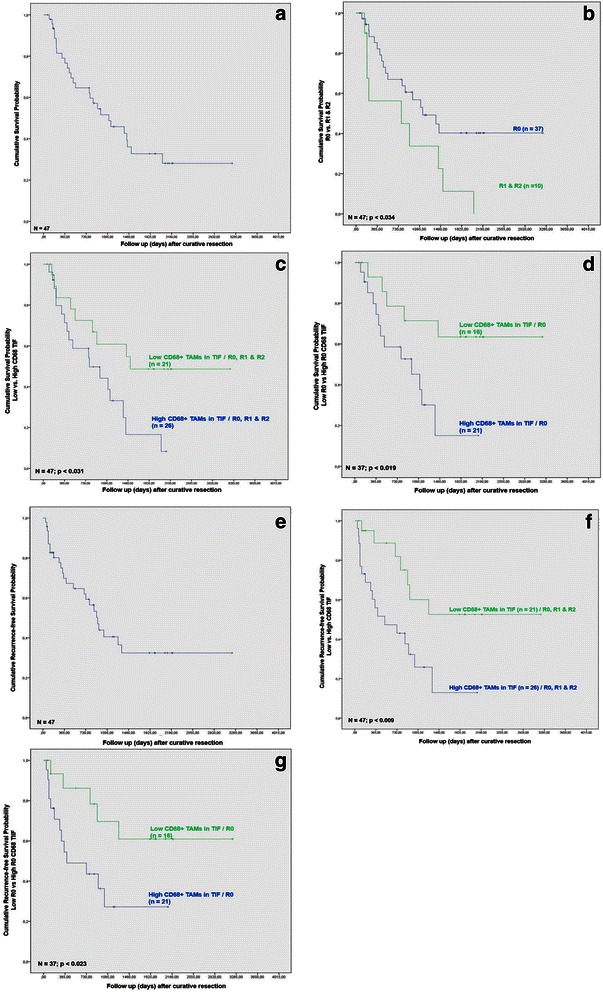
**a** Overall survival after surgery for hilar cholangiocarcinoma (*n* = 47). **b** Overall survival after surgery for hilar cholangiocarcinoma (*n* = 37) according to R status. **c** Overall survival after surgery referred to CD68-positve TAMs in the tumor infiltration front (TIF) regardless of R status (*n* = 47). **d** Overall survival after surgery referred to TAMs in the tumor infiltration front (TIF) after R0 resection (*n* = 37). **e** Recurrence-free survival of all patients after surgery for hilar cholangiocarcinoma (*n* = 47). **f** Recurrence-free survival after surgery correlated to TAMs in the tumor invasive front (TIF) regardless of R status (*n* = 47). **g** Recurrence-free survival correlated to TAMs in the tumor invasive front (TIF) following R0 surgery (*n* = 37)

The median follow-up after resection in all patients was 28 months (range 2.3 to 105.7 months). The overall 1-, 3- and 5-year survival of our cohort was 78.8, 51.4 and 31.7 %, respectively (Fig. 1a). The overall 1-, 3- and 5-year recurrence-free survival rates were 68.8, 40.1 and 31.3 %, respectively (Fig. 1e). 27 (57.4 %) of 47 patients died within the follow-up interval. 25 patients (53.2 %) developed tumor recurrence and 24 (51.0 %) patients died for recurrence of hilar cholangiocarcinoma. Local tumor recurrence was seen in 20 (42.6 %) patients, while 5 (10.6 %) patients developed distant metastases without manifestation of local recurrence (Table 1). In 6 (12.7 %) patients with local recurrence of hilar cholangiocarcinoma a metastatic spread of the tumor to distant sites was detected. 3 (6.3 %) patients died without evidence for tumor-related cause and had no signs of tumor recurrence at the time of death.

### Macrophage distribution in hilar cholangiocarcinoma

**Fig. 2:**
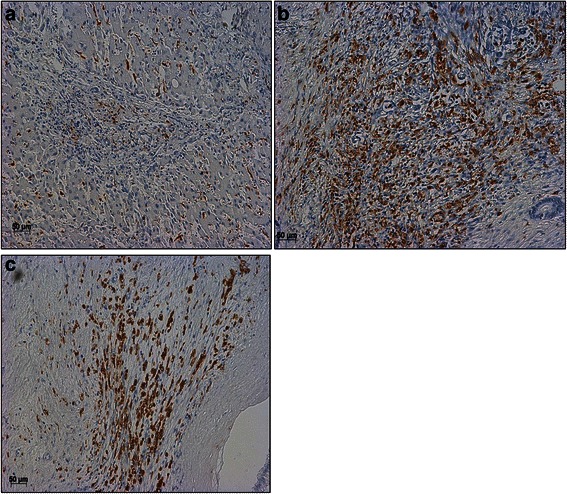
**a** CD68-positive macrophages in normal liver tissue. Normal liver parenchyma revealed a homogenous infiltration pattern with no preference for perivascular areas or clumping of TAMs. Original magnification: x 200; scale bar = 50 μm. **b** Hilar cholangiocarcinoma stained with mAb CD68 PG-M1 with a high abundance of TAMs (tumorous tissue “TT” score 3). Original magnification: x 200; scale bar = 50 μm. **c** Tumor infiltration front (TIF) positive for CD68-positive TAMs (TIF score 2 / high abundance / positive). Original magnification: x 200; scale bar = 50 μm

TAMs showed a vast homogenous distribution in tumor stroma and tumor parenchyma, TIF and perivascular areas, respectively (Fig. 2a-c). No clusters or ‘clumping’ of TAMs in tumor tissue has been observed. The infiltration patterns did not reveal any preference for the tumor perivascular areas. The majority of TAMs were apparently located in direct contact with or adjacent to tumor cells. Interestingly, a high density of TAMs was observed at TIF in 26 patients (TIF score 2/high density/positive), while rest of tumor specimens (*n* = 21) displayed only a scarce or complete absence of infiltrating TAMs (TIF scores 0 and 1/low density/negative) (Fig. 2c).

### The presence of tumor-infiltrating CD68-positive macrophages in tumor invasive front associates with tumor recurrence in patients with hilar cholangiocarcinoma

High abundance of macrophages in TIF correlated with a significantly increased overall tumor recurrence. 18 (69.2 %) of 26 patients in high TIF CD68 group displayed tumor recurrence (*ρ* = 0.015). In the low TIF CD68 group 7 (33.3 %) of 21 patients had recurrent disease. Moreover, patients with high levels of macrophages in TIF showed a significantly enhanced incidence of local tumor recurrence (*ρ* = 0.0001).

17 (65.4 %) of 26 patients in the high TIF CD68 group suffered a local tumor recurrence, whereas 3 (14.3 %) out of 21 patients in the low TIF CD68 group did not show local tumor recurrence (Table 2). After R0 resection (*n* = 37), a low CD68 abundance in TIF (‘R0 low CD68 TIF group’; *n* = 16) correlated with a significantly lower rate of disease recurrence, as well. 11 (68.8 %) and 15 (93.8 %) patients in the low CD68 TIF group did not develop overall or local tumor recurrence following R0 surgery (*ρ* = 0.064 and *ρ* = 0.001, respectively).

**Table 2:** Correlation of CD68-positiveTAMs in the tumor invasive front with clinicopathological characteristics of hilar cholangiocarcinoma

Clinicopathological analysis	ᅟ	ᅟ	ᅟ
Variable	high CD68	low CD68	p
No. of patients	26	21	
Gender			0.552
Male	13 (50 %)	10 (47.6 %)	
Female	13 (50 %)	11 (52.4 %)	
Patient age, years			0.448
≤60	14 (53.8 %)	12 (47.6 %)	
>60	12 (46.2 %)	11 (52.4 %)	
Histological differentiation			0.307
Well/moderate	21 (80.8 %)	19 (90.5 %)	
Poor	5 (19.2 %)	2 (9.5 %)	
Pathological T stage			0.413
T2	8 (30.8 %)	8 (38.1 %)	
T3	18 (67.9 %)	13 (61.9 %)	
Pathological N stage			0.307
Negative	19 (73.1 %)	13 (61.9 %)	
Positive	7 (26.9 %)	8 (38.1 %)	
Perineural sheath infiltration			0.390
Negative	3 (11.5 %)	1 (4.8 %)	
Positive	23 (88.5 %)	20 (95.2 %)	
Lymphangiosis carcinomatosa			0.576
Negative	12 (46.2 %)	10 (47.6 %)	
Positive	14 (53.8 %)	11 (52.4 %)	
Perivascular lymphangiosis			0.552
Negative	13 (50 %)	11 (52.4 %)	
Positive	13 (50 %)	10 (47.6 %)	
Vascular invasion			0.549
Negative	18 (69.2 %)	14 (66.6 %)	
Positive	8 (30.8 %)	7 (33.3 %)	
Tumor recurrence			0.015
Without	8 (30.8 %)	14 (66.7 %)	
With	18 (69.2 %)	7 (33.3 %)	
Local recurrence			0.001
Without	9 (34.6 %)	18 (85.7 %)	
With	17 (65.4 %)	3 (14.3 %)	
Distant metastases			0.390
Without	19 (73.1 %)	17 (81.7 %)	
With	7 (26.9 %)	4 (19.0 %)	

Other well established common prognostic factors were assessed, as well. 5 (19.2 %) of 26 patients in high TIF CD68 group had poor histologic tumor differentiation. In the low TIF CD68 group 2 (9.5 %) of 21 patients displayed poor histologic differentiation (*ρ* = 0.307). Related to angioinvasion, 8 (30.8 %) of 26 and 7 (33.3 %) of 21 patients in the high or low CD68 TIF group, respectively, showed a microscopic angioinvasion (*ρ* = 0.549). 18 (69.2 %) of 26 and 13 (61.9 %) of 21 patients in the high or low CD68 TIF group, respectively, showed a more advanced T stage (*ρ* = 0.413). Related to perineural sheet infiltration, 3 (11.5 %) of 26 and 1 (4.8 %) of 21 patients in the high or low CD68 TIF group, respectively, showed an absence of perineural sheet infiltration (ρ = 0.390).

### Prognostic significance of CD68-positive macrophages in hilar cholangiocarcinoma

Next, we analyzed whether TAMs in addition to other clinicopathological parameters predict survival after resection for hilar cholangiocarcinoma. In the univariate analysis R category, overall and local tumor recurrence, and low density of macrophages in TIF were associated with a statistically significant improvement of patient survival after resection (*ρ* = 0.039, *ρ* = 0.001, *ρ* = 0.001 and *ρ* = 0.036, respectively). As related to recurrence-free survival, in univariate analysis only low macrophages in TIF (*ρ* = 0.013) were associated with a statistically significant improvement.

In multivariate analysis presence of TAMs in TIF (related to recurrence-free survival for all patients, as well for R0 status only) and tumor recurrence were identified as independent prognostic factors for survival (all *ρ* < 0.05; Table 3). Other well established common prognostic factors, e. g. T stage and angioinvasion, did not exert prognostic significance in the current work.

**Table 3:** Univariate and multivariate analysis of prognostic factors in patients with hilar cholangiocarcinoma

Variable	Category	Odds ratio	p	Confidence interval
Univariate analysis				
Age	≤60/>60	1.588	0.239	0.735–3.430
Gender	male/female	1.287	0.517	0.600–2.761
perivascular lymphangiosis	negative/positive	0.734	0.430	0.340–1.583
R-Classification	negative/positive	2.325	0.039	1.042–5.189
Histologic differentiation	well or moderate/poor	2.141	0.103	0.857–5.344
Vascular invasion	negative/positive	1.285	0.569	0.542–3.045
pT	pT2/pT3	1.242	0.597	0.557–2.768
pN	negative/positive	0.535	0.124	0.242–1.186
Perineural sheet infiltration	negative/positive	0.289	0.224	0.039–2.137
Lymphangiosis carcinomatosa	negative/positive	0.835	0.651	0.381–1.827
CD68 + TAMs	high/low	0.977	0.951	0.457–2.088
CD68 + TAMs in TIF	high/low	0.322	0.013	0.132–0.784
Overall Tumor Recurrence	negative/positive	0.112	0.001	0.033–0.37
Distant Metastasis	negative/positive	0.528	0.111	0.240–1.159
Local Tumor Recurrence	negative/positive	0.183	0.001	0.076–0.441
Multivariate analysis				
R Classification	negative/positive	0.830	0.710	0.312–2.212
Overall Tumor Recurrence	negative/positive	0.172	0.024	0.037–0.794
Local Tumor Recurrence	negative/positive	0.544	0.432	0.119–2.486
CD68-positive TAMs in TIF	high/low	0.856	0.780	0.287–2.553
Multivariate analysis (Recurrence free survival)				
R0 & R1/2 surgery/*n* = 47				
R Classification	negative/positive	1.935	0.149	0.789–4.743
CD68-positive TAMs in TIF	high/low	0.324	0.014	0.132–0.796
R0 & R1/2 surgery/*n* = 47				
only R0 surgery/*n* = 37				
Histologic Differentiation	well or moderate/poor	2.549	0.109	0.811–8.012
CD68-positive TAMs in TIF	high/low	0.286	0.024	0.097–0.846
only R0 surgery/*n* = 37				

### Influence of macrophages on overall and recurrence-free survival in hilar cholangiocarcinoma

Based on the identification of TAMs in TIF as an independent prognostic factor in both the univariate and multivariate analysis, we investigated its effect on patients’ survival in hilar cholangiocarcinoma. The presence of TAMs in TIF affected patients’ survival after resection for hilar cholangiocarcinoma. Univariate analysis revealed a significantly better survival in the low TIF CD68 group (*ρ* = 0.013). The overall survival rates were 83.6 and 61.3 % at 1- and 3-year post surgery in comparison with 75.1 and 42.4 % at 1- and 3-year following resection for patients with low and high abundance of CD68-positive macrophages in TIF, respectively (Fig. 1c). Moreover, a low density of CD68-positive cells in TIF correlated with a significantly improved recurrence free survival. The 1-, 3-, and 5-year recurrence-free survival rates of patients with tumors that displayed low density of CD68-positive cells in TIF were 93.9, 59.8 and 51.8 %, whereas the 1-, 3-, and 5-year recurrence-free survival rates of patients with tumors with a high abundance of CD68-positive cells in TIF were 57.4, 26.2 and 12.1 %, respectively (Fig. 1f). The difference was statistically significant (*p* = 0.009). Following R0 resection, a low abundance of CD68-positive cells in TIF correlated with an improved overall and recurrence-free survival, as well (*p* = 0.19 and *p* = 0.023, respectively) (Fig. 1b, d, g).

Histologic differentiation and perineural sheet infiltration showed a distinct trend towards an improved overall survival, though without reaching statistical significance (*ρ* = 0.103 and *ρ* = 0.224, respectively). Overall survival and recurrence free survival of patients with hilar cholangiocarcinoma were significantly better in patients with low levels of TAMs in the area of TIF when compared to those with a high density of TAMs.

## Discussion

Analyzing immunoreactivity for CD68-positive TAMs in tumor samples from patients who underwent resection for hilar cholangiocarcinoma, we were able to show that (1.) a high abundance of CD68 in TIF is a risk factor for local recurrence and overall tumor recurrence, (2.) serves as an independent prognostic factor for survival, and (3.) translates into dismal overall as well as recurrence-free survival rates.

High density of TAMs in TIF was an independent negative prognostic factor for recurrence-free survival. Espinosa et al. demonstrated TAMs to correlate with increased microvessel proliferation and enhanced tumor angiogenesis [20]. Thus, reduced density of CD68 in TIF might mitigate tumor angiogenesis and improve survival in hilar cholangiocarcinoma. In the univariate analysis a negative-margin (R0) tumor resection correlated significantly with improved survival; however, this could not be confirmed in the multivariate analysis which is likely to be due to the small number of patients with positive-margin (R1, R2). The clinical characteristics and tumor specimens of the presented work were retrieved from a high volume center patient cohort with clinicopathologic features resembling other studies [21, 22]. In the multivariate analysis, only tumor recurrence and high abundance of CD68 in TIF irrespective of R-status proved to be independent prognostic indicators. A high abundance of CD68 in TIF was correlated not only with a higher risk of overall tumor recurrence but also with local tumor recurrence. These variables are well established indicators for unfavorable patient outcome and poor long term survival in hilar cholangiocarcinoma. Hereby, the stronger influence of TAMs in TIF on patient survival and recurrence-free survival compared to R classification may be caused by correlation with multiple unfavorable clinicopathologic characteristics in hilar cholangiocarcinoma.

The tumor mass is a multifaceted playground, where different cell types, including cancer cells, fibroblasts, endothelial and immune-competent cells are entangled into a constant interaction. Paradoxically, in the majority of human solid cancers, cells of the innate immune system, especially TAMs, were shown to favor tumor progression by, i.e., fostering metastasis and suppressing adaptive immunity. Yet, the lack of routine implementation of the experimental immunologic rationales as diagnostic or therapeutic concepts on a clinic daily basis is striking [23, 24]. In the present work we propose a well described and ubiquitous type of TAMs, in particular cells expressing CD68, as simple diagnostic tools prognosticating patient outcome in hilar cholangiocarcinoma.

In the current work TAMS in TIF were associated with survival and recurrence free survival and were identified as independent prognosticator. A rational clinical translation of these results suggests standardized utilization of TAMs as prognostic markers in the scope of pathological evaluation of resected specimens from patients suffering from hilar cholangiocarcinoma. This could be performed routinely in the scope of microscopical histological tumor evaluation as well as in standardized and reliable protocols on paraffin-embedded tumor specimens after staining with commercially available and commonly used antibodies against the macrophage marker CD68.

Another possibility for clinical utilization is the targeting of TAMs in tumors. The pro-tumor features of TAMs render these cell types attractive targets for biological anti-cancer therapies. To date, tumor progression is successfully treated by depletion of TAMs in preclinical animal models. Moreover, the combination of macrophage depleting agents with sorafenib, a potent inhibitor of tyrosine protein kinases (e.g. VEGF and platelet-derived growth factor receptor (PDGRF)), enhances significantly the efficacy of sorafenib alone in a xenograft model of hepatocellular carcinoma [25].

High recurrence rates after surgical resection for hilar cholangiocarcinoma imply an urgent need for reliable prognostic markers aiding the selection of appropriate patients who will benefit from radical surgical strategies. In particular, suitable patients for liver transplantation need to be cautiously selected, on grounds of a common donor organ shortage. CD68-positive TAMs and tumor necrosis may prove as valuable tools regarding patient selection. Emerging experimental studies reveal a potential role for selective in vivo visualization of circulating monocytes/macrophages and TAMs by magnetic resonance imaging (MRI) techniques allowing 3D spatial resolution up to 50 μm [26–31]. With a further development and clinical implementation of these imaging tools the evaluation of TAMs could offer an additional diagnostic modality for the patient selection for further treatment strategies.

However, drawbacks in the utilization of CD68 as a selection criterion prior surgery are to be considered, as well. To date, reliable clinical evaluation of these markers is only possible in resected tumor specimens following surgery. It should be also considered that tumor biopsies prior surgery will not provide representative tissue specimens and will not conform to the principles of a surgical approach using a ‘no-touch technique’ [32].

## Conclusions

In conclusion, in the current study we demonstrate for the first time that presence of CD68-positive TAMs in tumor invasive front correlates with tumor recurrence and serves as an independent prognostic factor for survival in hilar cholangiocarcinoma. Thus, TAMs may be crucially involved in the progression of this tumor entity and their utilization as a diagnostic tool or targeting TAMs, may deliver new therapeutic approaches in this cancer.

## Abbreviations

HCC: Hepatocellular carcinoma
H&E: Hematoxylin and eosin
mAb: Monoclonal antibody
PDGRF: Platelet-derived growth factor receptor
TBS: Tris buffered saline
TAMs: Tumor associated macrophages
TIF: Tumor infiltrating front
UICC: Union Internationale Contra Cancrum
VEGF: Vascular endothelial growth factor
